# Infant feeding bottle design, growth and behaviour: results from a randomised trial

**DOI:** 10.1186/1756-0500-5-150

**Published:** 2012-03-16

**Authors:** MS Fewtrell, K Kennedy, R Nicholl, A Khakoo, A Lucas

**Affiliations:** 1 MRC Childhood Nutrition Research Centre, UCL Institute of Child Health, London, UK; 2 Northwick Park Hospital, Harrow, London, UK; 3 The Hillingdon Hospital, Uxbridge, UK

**Keywords:** Infant feeding, bottle, breast, growth, milk intake, behaviour

## Abstract

**Background:**

Whether the design of an anti-vacuum infant feeding bottle influences infant milk intake, growth or behavior is unknown, and was the subject of this randomized trial.

**Methods:**

**Subjects**

63 (36 male) healthy, exclusively formula-fed term infants.

**Intervention**

Randomisation to use Bottle A (n = 31), one-way air valve: Philips Avent) versus Bottle B (n = 32), internal venting system: Dr Browns). 74 breast-fed reference infants were recruited, with randomisation (n = 24) to bottle A (n = 11) or B (n = 13) if bottle-feeding was subsequently introduced.

**Randomisation**

stratified by gender and parity; computer-based telephone randomisation by independent clinical trials unit.

**Setting**

Infant home.

**Primary outcome measure**

infant weight gain to 4 weeks.

**Secondary outcomes**

(i) milk intake (ii) infant behaviour measured at 2 weeks (validated 3-day diary); (iii) risk of infection; (iv) continuation of breastfeeding following introduction of mixed feeding.

**Results:**

**Number analysed for primary outcome**

Bottle A n = 29, Bottle B n = 25.

**Primary outcome**

There was no significant difference in weight gain between randomised groups (0-4 weeks Bottle A 0.74 (SD 1.2) SDS versus bottle B 0.51 (0.39), mean difference 0.23 (95% CI -0.31 to 0.77).

**Secondary outcomes**

Infants using bottle A had significantly less reported fussing (mean 46 versus 74 minutes/day, p < 0.05) than those using bottle B. There was no significant difference in any other outcome measure.

**Breast-fed reference group**

There were no significant differences in primary or secondary outcomes between breast-fed and formula fed infants. The likelyhood of breastfeeding at 3 months was not significantly different in infants subsequently randomised to bottle A or B.

**Conclusion:**

Bottle design may have short-term effects on infant behaviour which merit further investigation. No significant effects were seen on milk intake or growth; confidence in these findings is limited by the small sample size and this needs confirmation in a larger study.

**Trial registration:**

Clinical Trials.gov NCT00325208.

## Background

Modern infant feeding bottles vary substantially in appearance and design. Developments in bottle design have mostly focused on short-term infant behaviour; indeed, we have previously shown in a randomised trial that use of a feeding bottle with anti-vacuum features was associated with significantly less distressed vocalisation and a greater proportion of time spent awake and happy when compared to a conventional bottle without such features [1]. However, the possibility that feeding bottle design might affect infant milk intake and hence growth, or the risk of infection, has not previously been considered.

**Figure 1:**
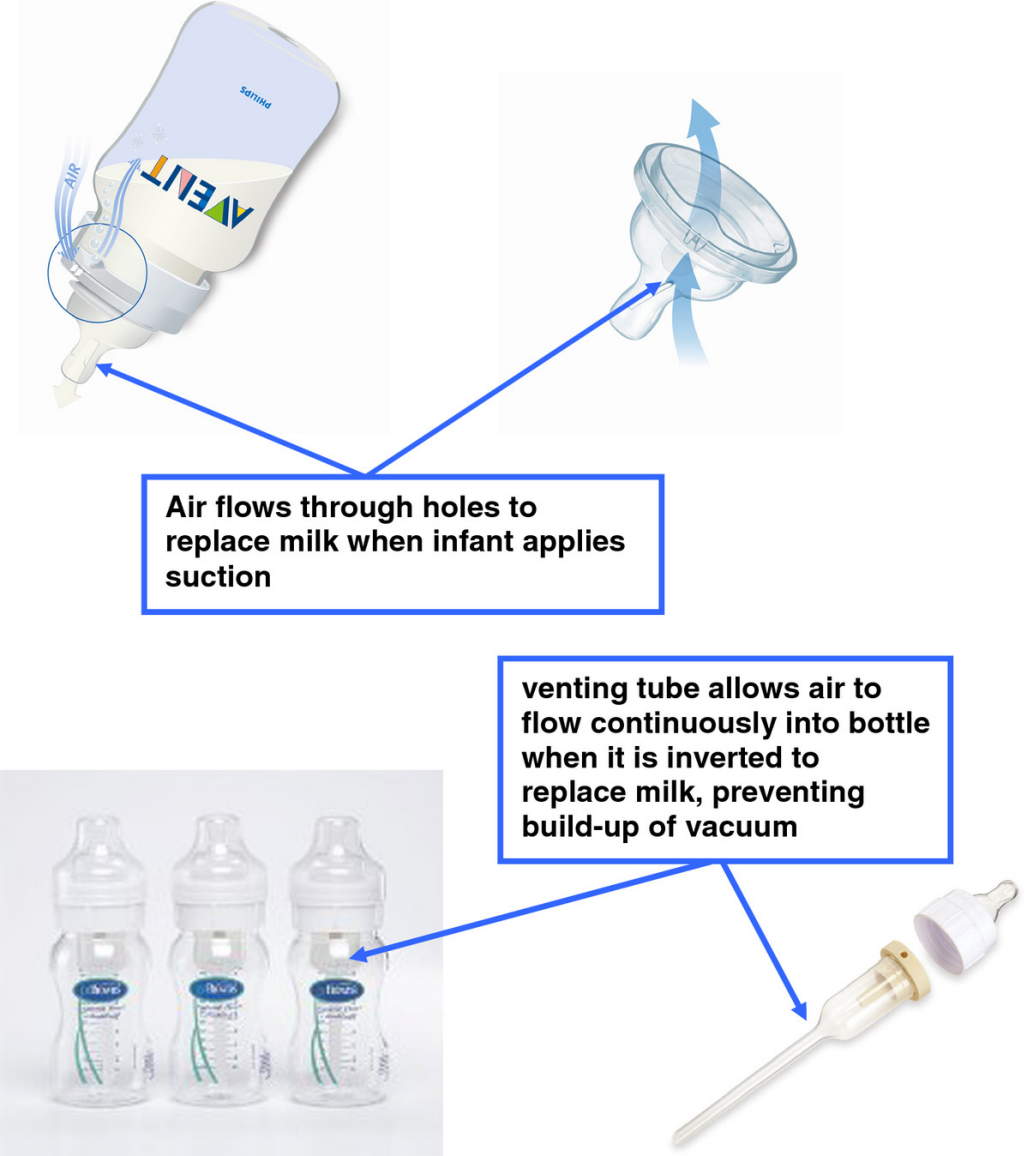
**Infant feeding bottles A and B, showing anti-vacuum features**. a) Bottle A; b) Bottle B.

In this study, we compared two commonly used infant feeding bottles with different anti-vacuum designs. The first bottle has a one-way air valve, allowing air to flow into the bottle to replace milk when the infant sucks, and may be described as 'partial anti-vacuum', (Bottle A; Philips Avent, Glemsford, Suffolk, UK; Figure 1a) whilst the second has an internal venting system which allows air to flow continuously into the bottle when it is inverted, and can be described as 'complete anti-vacuum' (Bottle B; Dr Browns, Handi-Craft, Missouri, USA; Figure 1b). We hypothesised that greater effort would be required to obtain milk from bottle A, and that this would result in (i) lower rates of milk intake and hence (ii) slower growth; and finally (iii) patterns of growth more similar to those of breast-fed infants.

The study also aimed to address secondary hypotheses related to infant behaviour and infection risk. Because of the complete venting design of bottle B, which prevents the build-up of any vacuum, we hypothesised that infants using this bottle would have less colic, be more settled, and that the reduced requirement for suction might prevent the build-up of negative pressure in the infant's mouth and pharynx [2] which could in turn reduce the build up of fluid in the middle ear and reduce the incidence of ear infections. Conversely, related to the greater complexity of parts of bottle B, we hypothesised that mothers would award lower scores on a range of consumer characteristics, particularly those relating to assembly and cleaning, which might in turn be reflected in an increased risk of gastrointestinal infection.

## Methods

Healthy term singleton infants (37 weeks completed gestation, birthweight > 2.5 kg) were recruited from the post-natal wards at 2 hospitals (The Hillingdon Hospital, Uxbridge, UK and Northwick Park Hospital, Harrow, UK) between June 2006 and November 2007. Exclusion criteria were: birthweight < 2.5 kg, gestational age < 37 weeks, any congenital malformation likely to affect growth, requirement for neonatal intensive care, or mother unable to speak English (necessary because of the questionnaires and diaries used). Ethical approval was granted by the Institute of Child Health Research Ethics Committee. The trial was registered with Clinical Trials.gov (NCT00325208).

Mothers were given an information sheet whilst they were in hospital and asked for permission to be contacted at home soon after discharge to determine if they wished to participate. No attempt was made to influence feeding mode - indeed all mothers were encouraged and supported to breast-feed. If the mother was exclusively bottle-feeding when contacted at home, she was asked for consent to be randomised to use either bottle A or B. If breast-feeding fully, she was recruited into the reference group. Mixed feeders were not recruited into the study, because of the potential for discouraging breastfeeding. The higher than anticipated rate of mixed feeding in the early post-natal period led to difficulties in recruiting sufficient exclusively bottle-fed infants from the post-natal wards, and some infants were also recruited via health centres and family doctors' surgeries.

### Randomisation

After checking eligibility and obtaining written informed consent, the research nurse telephoned the randomisation centre based at the MRC Clinical Trials Unit (CTU), London. Standardised questions were asked by the centre using an agreed database to confirm eligibility and the randomisation assignment was given. Randomisation of formula-fed infants was stratified by parity and by infant gender. Confirmation of enrolment and randomised assignment was sent by e-mail and fax to the study centre. Breast-fed infants were registered with the CTU at the point of enrolment and assigned a study number. If a breastfeeding mother later started using a bottle and consented to randomisation, the research nurse telephoned the CTU to obtain the randomised assignment. Confirmation of the assignment was sent by fax and e-mail to the study centre.

All formula-feeding mothers were offered a supply of free infant formula (Farley's First Milk (Heinz plc, Kendal, UK) packed in plain research tins without the company name or logos) until the infant was 6 months old, in an attempt to minimise any effect of different infant formulas on growth; however, many mothers wished to choose their own formula and this did not result in exclusion from the trial. Mothers were asked to use the randomised bottle for at least the first 4 weeks of the study. Beyond this time point they were encouraged to continue using the randomised bottle until they felt that their infant was ready to progress to a beaker or cup. Mothers were initially provided with 6 bottles and newborn teats. At the 4 week visit they were provided with the next size teat. We made no attempt to influence the method used for sterilisation of feeding equipment, although the need for good hygiene was emphasised at all times.

Exclusive breastfeeding for 6 months was recommended as per current guidelines from the UK Department of Health. However, if at any stage a breast-feeding mother expressed the intention of introducing a bottle to feed her infant expressed breast milk or infant formula, she was asked for permission to be randomised to use bottle A or bottle B. If she agreed, written informed consent was obtained. The mother was provided with a supply of bottles, but free formula milk was not provided as this might undermine continued breastfeeding.

All outcomes measures were recorded during home visits at enrolment, 2, 3 and 4 weeks and subsequently at 3 month follow-up. The primary outcome measure was infant weight gain from enrolment to 4 weeks. Secondary outcome measures related to (i) growth (length and head circumference gain, milk intake from enrolment to 2 weeks); (ii) infant behaviour at 2 weeks of age; (iii) infection (ear and gastrointestinal infections up to 3 months); (iv) mother's opinion of the bottle at 4 weeks; and (v) proportion breast-fed infants subsequently randomised to bottle A or B who were receiving any breastfeeding and exclusive breastfeeding at 3 months.

Baseline data were collected on social and demographic factors, and on the mother's pregnancy and delivery. Infant anthropometry was recorded at each home visit. Weight was obtained using digital scales with the infant naked. Length was measured using a rollameter and head circumference and mid upper arm circumference with a non-stretchable tape. Measurements were performed in triplicate and the mean value used for analyses. Weight, length and head circumference were converted to SD scores using UK90 reference data [3].

### Milk intake

Bottle-feeding mothers were asked to keep a diary each day between randomisation and the 2 week visit, recording the number of feeds, the weight of milk taken at each feed and the time taken for the feed. Scales were provided for the mother to weigh feeding bottles before and after each feed.

### Infant behavior

At 2 weeks, mothers were asked to complete a validated infant behaviour diary [4-6]. recording the time spent in 6 different behavioural states (colic, crying, fussing, awake & happy, feeding and asleep) over a 3 day period. Clear definitions for each state were provided; for example, the definition of 'fussing' was 'baby irritable and unsettled, may be vocalising but not continuously crying'; that for 'crying' was 'periods of prolonged, distressed vocalisation; and that for 'colic' was 'bouts of intense, unsoothable crying and other behaviour, possibly due to stomach or bowel pain'. Each 24 hour period was divided into 15 minute periods, and the parent was asked to record the infant's behavioural state for each period by shading the appropriate box on a grid. The mean number of minutes spent in each behavioural state over the 3 day reporting period was calculated for each infant by the research nurse. The behaviour diary has been extensively validated and used successfully by members of the collaborative team in previous studies [5].

### Ear and gastrointestinal infections

At each visit, questionnaires were used to record details of infant feeding history and general health including specifically the number of ear infections and gastrointestinal infections. A diagnosis of ear or chest infection was only accepted if it had been diagnosed by the family doctor and treated with antibiotics.

### Mothers' opinions of bottle characteristics

At the 4 week visit, a questionnaire was used to assess mothers' opinions of the two bottles for four parameters: ease of assembly, cleaning, appearance and how comfortable the bottle was to hold. Each parameter was rated on an analogue scale with 1 as most favourable, 4 neutral and 7 as least favourable. Their scores were recoded into 3 categories for analysis (1-3, 4 and 5-7) representing 'above average', 'neutral' and 'below average'.

### Sample size and recruitment

A sample size of 76 infants per randomised group was planned to allow detection of 0.5SD difference in outcome measures between bottle groups for main study, allowing for an interim analysis when all infants completed 1 month in the study. We also aimed to recruit 76 breast-fed infants as a reference group.

### Statistical analyses

The primary analysis was performed on an intention-to-treat basis, comparing data from the randomised bottle groups using Student's *t*-test or chi-square test (or non-parametric statistics as appropriate). Secondary analyses were performed where appropriate for subjects who remained on their assigned bottle at the time of measurement. Data from the breast-fed reference group were compared to those from the two formula groups by analysis of variance (ANOVA). Dunnett's post-hoc test was used to compare individual formula groups against the breast-fed group where the ANOVA was significant. General linear models were used to compare groups after adjusting for relevant confounding factors. Secondary analyses included only those infants who were still breast-feeding at the time of the outcome assessment.

## Results

### Comparison of randomised groups

#### Study population

**Figure 2:**
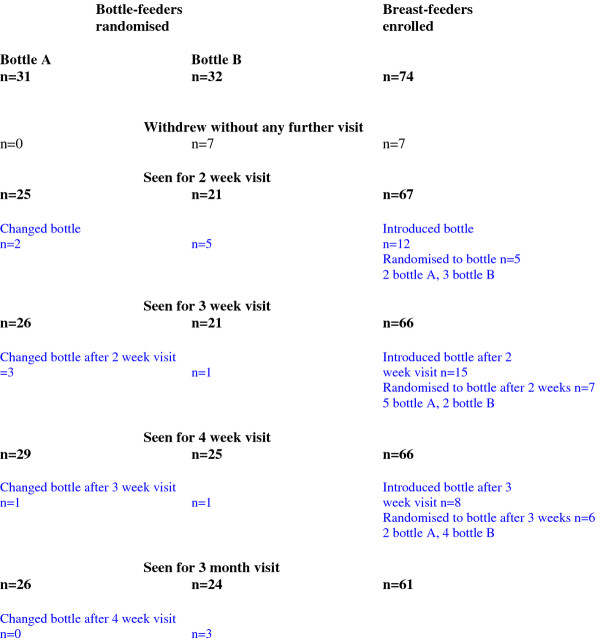
**Flow of subjects through study**.

Changes in UK infant feeding practices, in particular the fact that more mothers were choosing to mix breast and bottle-feeding rather than exclusively bottle feeding early in the post-natal period, made the target sample size unachievable within the time-frame and budget for the study. Figure 2 shows the flow of subjects through the study. 31 infants were randomised to bottle A and 32 infants to bottle B. 29 infants randomised to bottle A and 25 randomised to bottle B were seen at 4 weeks; of these, 13 infants had changed bottle (6 from group A (5 to bottle B, one unknown) and 7 from group B (3 to bottle A, 3 to other brands, one not known)); 3 mothers using bottle A reported changing because the infant had colic, 5 bottle B mothers reported that the bottle leaked and 1 that they took too long to clean. Other mothers did not give a specific reason for changing bottle. Specific reasons given for changing bottleOne infant (randomised to bottle A) was recruited at 22 days, following the decision to increase the age-range for recruitment. However, in practice no other infants were recruited later than 15 days. Since this single infant was an outlier on initial inspection of the data (not surprising as his baseline data were recorded at the same time as 2 or 3 week data for other infants), the decision was taken to omit the infant from the analyses. The mother had also changed from the randomised bottle to use bottle B shortly after randomisation and would therefore have been excluded from any 'secondary' analysis on this basis. Twenty-six infants randomised to bottle B and 24 infants randomised to bottle B were seen for follow-up at 3 months.

Baseline characteristics of the two groups are shown in Table 1. There were no significant differences between randomised groups. The proportion of non-smoking mothers was lower, although not significantly so, in subjects randomised to bottle B.

**Table 1:** Baseline data (mean (SD) or n (%)) for randomised groups and the breast-fed reference group

	Bottle A	Bottle B	Breast-fed
	n = 31	n = 32	n = 74

Birth weight (g)	3518 (441)	3322 (381)	3363 (407)

Gestational age (weeks)	39.9 (1.4)	39.4 (2.5)	39.7 (1.2)

Boys n (%)	18 (58)	18 (56)	39 (53)

Primips n (%)	9 (29)	12 (39)	31 (43)

Vaginal delivery n (%)	18 (58%)	20 (65%)	58 (81%)

Social code 1 or 2 n (%)	7 (24%)	5 (17%)	52 (76%)*

Ethnic group of infant n (%)			

White	18 (55%)	20 (66%)	32 (45%)

Asian	6 (19%)	6 (20%)	21 (29%)

Black	2 (7%)	3 (11%)	8 (11%)

Mother's age (yrs)	29.0 (6.5)	27.8 (7.6)	31.7 (4.8)*

*% non-smokers*			

*Mother*			

1st trimester	86%	65%	95%**

2nd/3 rd trimester	79%	68%	97%**

Current	83%	65%	95%**

*Father*	60%	58%	78%**

* p < 0.05 breast-fed group compared to bottle-fed groups (ANOVA)

** p = 0.001 chi square for the proportion of non-smokers according to the different stages of gestation and feeding group; and for smoking/non-smoking in fathers.

Social code consists of 5 categories; 1 or 2 indicates that the breadwinner is in a professional or managerial position

##### Primary outcome measure

Infant weight gain (as change in absolute weight or in weight SD score) did not differ significantly between groups from enrolment to 4 weeks (Bottle A 785 (205)g versus Bottle B 875 (290)g, mean difference -90 (95% CI -230 to 50)g: Bottle A 0.74 (SD 1.2) versus bottle B 0.51 (0.39) mean difference 0.23 (95% CI -0.31 to 0.77). The findings were unchanged when weight gain over the period from birth to 4 weeks was analysed and differences between groups in weight gain over other study periods were also not significant. Absolute weight gain over the different periods, adjusted for baseline weight, gestational age, sex and the number of days between visits was not significantly different between groups.

#### Secondary outcome measures

*1. Growth and milk intake*: Infant length and head circumferences at randomisation and follow-up visits (Table 2) were not significantly different between the randomised groups. Mean daily milk intake (ml/kg/day) and the number of feeds per day during the first 2 weeks did not show any significant group differences for individual days or when averaged over several days; for example, mean milk intake per day between days 10 and 15 was 163 (31) ml/kg/day for Bottle A infants and 164 ml/kg/day (18) for Bottle B; p = 0.9). The results from the intention-to-treat analysis did not differ from the analysis excluding infants who changed feeding bottle.

**Table 2:** Infant anthropometry at baseline, 2 and 4 weeks, and 3 months for randomised groups and the breast-fed reference group (results are mean(SD))

	Avent	Dr Brown	Difference (95% CI)	p	Breast-fed
	n = 31	n = 32	n = 74		

Birth weight (g)	3518 (441)	3322 (381)	196 (-15 - 407)	0.07	3363 (407)

Birthweight SDS	0.15 (0.92)	-0.24 (0.78)	0.39 (-0.05 - 0.83)	0.08	-0.13 (0.90)

**Enrolment**					

Age at randomisation or enrolment (days)	7.5 (3.7)	7.1 (3.1)			7.5 (2.1)

Weight (g)	3559 (537)	3340 (375)	219 (-16.8 - 454)	0.07	3352 (393)

Weight SD	-0.40 (0.93)	-0.73 (0.74)	0.33 (-0.1 - 0.76)	0.1	-0.71 (0.86)

Length (cm)	51.1 (2.6)	50.3 (2.0)	0.81 (-0.35 - 2.0)	0.2	50.5 (2.5)

Length SD	-0.33 (1.15)	-0.62 (0.99)	0.29 (-0.25 - 0.83)	0.3	-0.53 (1.28)

OFC (cm)	35.8 (1.5)	35.1 (1.5)	0.73 (-0.02 - 1.5)	0.06	35.4 (1.4)

OFC SD	0.22 (1.07)	-0.28 (1.1)	0.50 (-0.04 - 1.04)	0.07	-0.07 (1.2)

**2 weeks**	**n = 24**	**n = 20**			**n = 66**

Age (days)	14.8 (1.7)	15.2 (2.6)	14.7 (2.1)		

Weight (g)	3776 (509)	3575 (311)	201 (-62 - 465)	0.1	3635 (385)

Weight SD	-0.35 (0.91)	-0.53 (0.76)	0.18 (-0.34 - 0.69)	0.5	-0.56 (0.80)

Length (cm)	52.3 (2.3)	51.2 (1.9)	1.14 (-0.18 - 2.5)	0.09	52.3 (2.4)

Length SD	-0.13 (1.08)	-0.58 (0.90)	0.39 (-0.23 - 1.0)	0.2	-0.06 (1.23)

OFC (cm)	36.5 (1.4)	35.8 (0.95)	0.72 (-0.024 - 1.5)	0.06	36.2 (1.0)

OFC SD	0.31 (1.0)	-0.20 (0.76)	0.44 (-0.11 - 0.99)	0.1	0.12 (0.89)

**4 weeks**	**n = 27**	**n = 22**			**n = 65**

Age (days)	29.0 (2.3)	29.8 (3.6)			29.1 (2.9)

Weight (g)	4294 (503)	4181 (380)	151 (-111 - 414)	0.3	4244 (451)

Weight SD	0.33 (1.28)	-0.27 (0.73)	0.6 (0.016 - 1.19)	0.04	-0.14 (0.87)

Length (cm)	54.0 (2.2)	53.4 (2.0)	0.71 (-0.5 - 1.9)	0.2	54.4 (2.0)

Length SD	0.27 (1.24)	-0.33 (1.01)	0.6 (-0.04 - 1.2)	0.07	0.16 (0.99)

OFC (cm)	37.6 (1.28)	37.4 (0.9)	0.32 (-0.31 - 0.9)	0.3	37.6 (1.5)

OFC SD	0.80 (1.34)	0.32 (1.1)	0.48 (-0.24 - 1.2)	0.2	0.22 (0.84)

**3 months**	**n = 26**	**n = 23**			**n = 61**

Age (yrs)	0.25 (0.04)	0.24 (0.03)			0.25 (0.02)

Weight (kg)	6.30 (0.78)	6.00 (0.68)	0.31 (-0.11 - 0.73)	0.1	6.04 (0.70)

Weight SDS	0.36 (1.0)	0.09 (0.94)	0.26 (-0.29 - 0.82)	0.3	0.04 (1.07)

Length (cm)	60.9 (2.2)	59.9 (2.0)	0.96 (-0.24 - 2.2)	0.1	60.9 (2.7)

Length SDS	0.25 (1.0)	-0.07 (1.0)	0.33 (-0.23 - 0.88)	0.2	0.27 (1.3)

OFC (cm)	41.0 (1.6)	40.5 (1.2)	0.54 (-0.29 - 1.4)	0.2	40.5 (1.1)

OFC SDS	0.16 (1.1)	-0.11 (0.9)	0.27 (-0.32 - 0.86)	0.4	-0.25 (1.0)

*2. Infant behaviour*: Results from the infant behaviour diaries are shown in Table 3. The median number of minutes per day spent in each of the six different behavioural states is shown. The three 'distressed vocalisations' - fussing, crying and colic - in total accounted for 6.6% of the day (approximately 95 minutes per day, with 'fussing' the commonest single distress behaviour, accounting for 4.5% of the day overall or approximately 65 minutes). Infants using bottle A were reported to spend less time 'fussing' than those using bottle B (median 40 (25^th^, 75^th ^centiles 9,73) versus 85 (40,109) minutes). When analysed separately for the periods 'day' (6 am to 6 pm) and 'night (6 pm to 6 am), reduced 'fussing' was reported in bottle A infants during both periods, although the difference was greater at night (Day: 25 (0,35) minutes versus 39 (13,55) minutes, p = 0.2: Night 13 (4,33) minutes versus 33 (15,61) minutes, p < 0.05). The amount or proportion of time spent in other individual behaviours, including colic, was not significantly different between groups. Excluding infants who had changed bottle at the time of the diary did not alter the findings although the difference in 'fussing' reported between groups was slightly greater (Bottle A median (25th, 75th centiles) 40 (3,71) minutes versus Bottle B 85 (40,120) minutes; p = 0.04).

**Table 3:** Results of infant behaviour diaries collected at the 2 week visit, according to randomised group (results are median (25th, 75th centiles) minutes per day spent in each behavioural state)

	Bottle A	Bottle B		Breast	
	**n = 22**	**n = 18**	p*	**n = 47**	p**

*Minutes per day*					

Crying	0 (0,16)	1 (0,19)	0.8	0 (0,37)	0.9

Fussing	40 (9,73)	85 (40,109)	0.045	65 (25,105)	0.13

Colic	0 (0,16)	0 (0,16)	0.8	0 (0,10)	1.0

Awake and happy	215 (156,279)	182 (130,308)	0.5	203 (150,285)	0.7

Asleep	914 (808,978)	933 (815,1005)	0.7	861 (783,935)	0.2

Feeding	235 (173,278)	195 (128,282)	0.4	245 (210,304)	0.1

* Mann-Whitney bottle A versus bottle B

** Kruskal-Wallis

*3. Infections*: The number of infants with reported gastroenteritis or ear infections is shown in Table 4. No statistically significant differences were seen although the small sample size limits the ability to exclude differences between groups.

**Table 4:** Reported gastroenteritis and ear infections according to study group (n(%))

	Bottle A	Bottle B	Breast-fed
***Gastroenteritis***			

First 4 weeks*	1 (3)	0	4 (6)

4 weeks-3 mo**	3 (11)	2 (8)	2 (3)

***Ear infections***			

First 4 weeks*	0	1 (4)	0

4 weeks-3 months**	2 (7)	0	1 (2)

* percentages relate to all infants seen at 4 weeks

** percentages relate to all infants seen at 3 months

**Figure 3:**
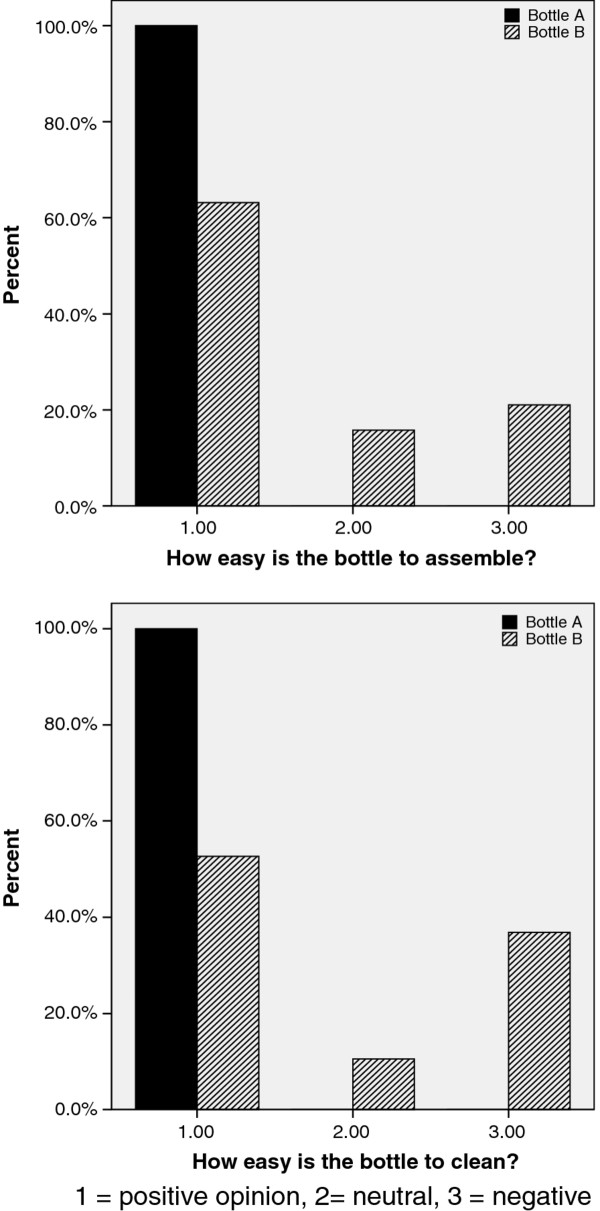
**Mothers' opinions of feeding bottles**.

*4. Mothers' opinions*: Mothers rated their assigned bottle for four characteristics on a 7 point analogue scale (Figure 3). Their scores were recoded into 3 categories for analysis (1-3, 4 and 5-7) representing 'above average', 'neutral' and 'below average'. Bottle A mothers awarded significantly better scores for two parameters: ease of assembly and ease of cleaning; 100% of Bottle A mothers awarded 'above average' scores for both questions compared to 63% and 53% of bottle B mothers for 'ease of assembly' (p = 0.009) and 'ease of cleaning' (p = 0.002), respectively. The difference between bottle groups was more significant when the analysis was confined to mothers who had not changed bottle during the first 4 weeks.

### Comparison of bottle-fed infants with the breast-fed reference group

#### Study population

74 breast-fed infants were enrolled. 7 infants withdrew before the 2 week visit and an additional infant between 2 and 3 weeks, leaving 66 infants for the 3 and 4 week visits. 61 infants were seen at 3 months. Baseline characteristics of the breast-fed infants are shown in Table 1. Compared to the bottle-fed infants, a greater proportion of breast-fed infants were classified as having higher socio-economic status (social codes 1 and 2; p < 0.001). Breast-feeding mothers were significantly older than bottle-feeding mothers (p = 0.008; post-hoc breast v bottle A p = 0.14, breast v bottle B p = 0.006), and the proportion of non-smoking mothers and fathers was higher in the breast-fed group (p < 0.05).

The total number of breast-fed infants randomised to use a bottle by 3 months was 24; 11 to bottle A and 13 to bottle B. 18 infants were randomised during the first 4 weeks and a further 6 between 4 and 12 weeks.

##### Primary outcome

Weight gain as SD scores from enrolment to 4 weeks (0.57 (SD 0.51)) did not differ significantly between breast-fed and bottle-fed infants. Length and head circumference did not differ significantly between breast-fed and bottle-fed infants at any time-point.

##### Infant behaviour

There were no significant differences between breast-fed infants and bottle-fed infants (Table 3).

##### Infection

During the first 4 weeks, 4 breast-fed infants were reported to have had 'gastroenteritis'. The number of reported infections was small (Table 4) with no differences between breast-fed and bottle-fed infants. The findings were unchanged in secondary analyses including only those infants still using the assigned bottle or still exclusively breast-feeding at the follow-up visit.

##### 4. Duration of reported breastfeeding in breast-fed infants randomised to use either bottle A or bottle B up to the 3 month visit

Reported rates of 'any' breastfeeding at 3 months were 91% in breast-fed infants using bottle A versus 62% for breast-fed infants using bottle B (p = 0.2) with 18% versus 31% reporting 'exclusive' breastfeeding (no formula but could include solid foods; (p = 0.6).

## Discussion

Our study, using two infant feeding bottles with different anti-vacuum features, is the first to evaluate experimentally whether the design of an infant feeding bottle influences a range of infant health and behaviour outcomes. However, our ability to draw conclusions from the study, particularly relating to growth patterns and infections, was limited by the small sample size. Because of positive changes in infant feeding patterns in the UK, in particular the greater proportion of mothers choosing to combine breastfeeding and bottle feeding in the early post-natal period rather than exclusively bottle-feeding, we were unable to achieve our planned sample size, and the actual sample size, with 30 infants per group, would only allow us to detect a 0.7SD difference in outcome measures between randomised groups. We may therefore have missed a smaller effect of biological significance. We did not recruit mixed fed infants because of the potential for discouraging breast-feeding, and hence our pool of eligible infants was lower than had been anticipated based on previous data.

### Milk intake and growth

Our primary hypothesis was that feeding bottle design would influence infant milk intake and hence growth. This is an important issue in view of the increasing evidence that rapid growth in early infancy is associated with an increased later risk of cardiovascular disease and obesity [7], which has led to a greater focus on the factors influencing infant growth, in particular the type and composition of milk. Breast-fed infants have lower milk and nutrient intakes during the early post-natal period than infants who receive infant formula. It is likely that, due to the supply and demand nature of breast-feeding, these infants are able to regulate their intake more effectively and signal satiety. In contrast, bottle-fed infants have less opportunity to influence their milk intake and may therefore be effectively overfed. The possibility that the design of an infant feeding bottle might influence the availability of milk, and hence infant nutrient intake and growth, has not previously been investigated.

We hypothesised that milk would be more easily available to infants using the completely vented bottle B, resulting in greater milk intake and faster weight gain. Our study did not confirm this primary hypothesis, although our analyses were hampered by the small sample size, which resulted in some baseline imbalance in anthropometry; bottle A infants tended to be heavier at birth and remained so on average throughout the study. This imbalance is particularly problematic in a study focussing on early growth, since the potential for catch-up growth is related to size at birth. Nevertheless there were no consistent trends towards more rapid growth in infants using bottle B and the patterns of weight gain expressed in SD scores were remarkably similar in all three groups of infants.

### Infant behaviour

We hypothesised that the complete venting design of Bottle B, which prevents the build-up of any vacuum inside the bottle, would result in a lower incidence of colic. Although the biological nature of excessive infant crying and colic is much debated, from a pragmatic perspective, these behaviours are well recognised to be a major area of concern for parents and to result in a substantial use of health service resources [6,8], and it is important to identify simple aspects of infant care that might prevent or reduce them. We recorded infant behaviour at 2 weeks of age since, in a previous study, this was identified as a peak time for distressed behaviours in bottle-fed infants [4].

In order to measure infant behaviour as objectively as possible without undue demands on the family, we used a 3 day diary which has previously been validated against concurrent audiorecordings of infant vocalisation [4-6]. Infant distress vocalisations - fussing, crying and colic - were clearly defined in the diaries and mothers appear to have no problem in understanding these definitions. Our results showed that the three distress vocalisations - fussing, crying and colic - together accounted for approximately 6.6% of the day - around 95 minutes. 'Fussing' was the single most common form of infant distress behaviour - accounting for 4.5% or 65 minutes per day - and was significantly influenced by bottle type; infants using bottle A were reported to fuss for on average 45 minutes per day less than those using bottle B. The difference was present throughout both the day and night, but slightly greater at night. This type of behaviour, often described by the mother as the infant being 'unsettled', can be extremely trying to parents as an underlying reason is frequently not apparent, and this finding is therefore likely to be of practical importance to mothers and carers.

In contrast to fussing, we found no significant differences between randomised groups in the duration of reported crying or colic. The proportion of infants reported to have at least one episode of colic during the 3 day period was around 30% in both bottle groups and the breast-fed group, with similar reported durations of colic in affected infants from all groups. In a previous study [3], we reported a lower duration of colic in infants using the partial anti-vacuum bottle A compared to those using a standard bottle with no anti-vacuum features; infants using bottle A in that study had values similar to a group of breast-fed reference infants. The lack of difference in reported colic between infants using the two feeding bottles in our current study - both of which have anti-vacuum features - may reflect the lack of power of our current study to detect differences in this relatively infrequent outcome. Alternatively, it may suggest that the complete venting design does not provide any greater advantage than the partial anti-vacuum design for this outcome. Nevertheless, the finding that the design of a feeding bottle has measurable effects on troublesome infant behaviours now in two different studies is important.

Compared to bottle-fed infants, breast-fed infants showed a trend towards shorter sleep times and greater feed times, with fussing times similar to those reported for infants using Bottle B and higher than for those using Bottle A. These differences are not unexpected, as breastfeeding mothers were feeding on demand and the diary was completed at an age (2 weeks) when breastfeeding is still being established. All differences between groups were more significant in secondary analyses, including infants who remained on their assigned bottle or who were still exclusively breast-feeding at the 2 week study visit.

#### Infections

We aimed to test the hypothesis that the complete venting design of bottle B would result in a reduced incidence of ear infections. It has been suggested that the reduced requirement for suction with bottle B might prevent the build-up of negative pressure in the infant's mouth and pharynx [2] which could in turn reduce the build up of fluid in the middle ear and influence the propensity to ear infections. In fact, the incidence of ear infections treated with antibiotics was low in our study compared to the recently reported figure of 44% for the prevalence of a first episode of acute otitis media in infants under 1 year of age [9]. There were no consistent differences in the reported incidence of ear infections between randomised groups, nor between breast-fed and bottle-fed infants. However, the small sample size limits our ability to draw conclusions.

We also hypothesised that the greater complexity of parts in Bottle B might compromise cleaning, resulting in an increased incidence of gastroenteritis. However, whilst mothers using bottle A awarded significantly higher scores for 'cleaning' and 'assembly' characteristics, with 100% awarding the highest scores in these categories, this was not reflected in detectable differences in gastroenteritis between groups, although this analysis was also limited by the small sample size. Our limited (unpublished) data, with microbiological testing of bottles following normal cleaning and 'sterilisation' routines by mothers shows a significant rate of isolation of potentially enteropathogenic organisms from bottles of both types, but with an excess in Bottle B. Anecdotally, the research nurses who collected the bottles observed that mothers frequently failed to wash their hands before assembling the bottles that were provided for testing. It is possible that the subsequent use of hot water to reconstitute formula may re-sterilise these bottles so that the infant is not adversely affected. Nevertheless, these findings re-emphasise the importance of good hygiene and, in particular, hand-washing prior to assembling feeding bottles.

## Conclusion

Due to the small sample size, we were unable to exclude effects of infant feeding bottle design on infant growth patterns or infection rates. However, our study showed that the design of an infant feeding bottle has effects on short-term infant behaviour, and influences parental opinions on aspects such as cleaning and assembly. These issues are likely to be of importance to parents and carers, and are therefore relevant for health professionals advising parents on the care of newborn infants.

## Competing interests

MF and AL have received research grants from the manufacturer of one of the feeding bottles used in this study and have performed advisory work for this company (Philips Avent). They have also received research funding and performed Consultancy and Advisory work for manufacturers of infant feeding products. Both are (and have been previously) members of National and International Committees and Expert groups advising on different aspects of infant nutrition. KK, RN and AK have no conflict of interest to declare with respect to this study.

## Authors' contributions

MF conceived and designed the study, performed the data analysis and interpretation and was responsible for drafting and revising the manuscript; KK was involved in study design, data collection and analysis, and revising the manuscript; RN and AK contributed to study design and data collection and assisted in revising the manuscript; AA was involved in conceiving and designing the project, data interpretation and revising the manuscript critically for important intellectual content. All authors have given final approval of the version to be published.

## Role of funding source and sponsor

The study was supported by a research donation from Philips AVENT, who also provided the study bottles. The funder had no involvement in the conduct of the study, data analysis or drafting of the manuscript. The study was sponsored by UCL Institute of Child Health, London.

## Funding source

Research grant from Philips AVENT.

## Study sponsor

UCL Institute of Child Health, London.
